# Post-COVID care delivery: The experience from an Irish tertiary centre’s post-COVID clinic

**DOI:** 10.1371/journal.pone.0289245

**Published:** 2023-08-11

**Authors:** Aoife Heeney, Stephen P. Connolly, Rachel Dillon, Aisling O’Donnell, Tara McSweeney, Brendan O’Kelly, Aoife G. Cotter, Gerard Sheehan, John S. Lambert, Eavan G. Muldoon, Tara McGinty

**Affiliations:** 1 Department of Infectious Diseases, Mater Misericordiae University Hospital, Dublin 7, Ireland; 2 School of Medicine, University College Dublin, Dublin 4, Ireland; Universidad Nacional Hermilio Valdizan Escuela Academico Profesional de Medicina Humana, PERU

## Abstract

**Background:**

The long-term effects of SARS-CoV-2 infection and optimal follow-up approach are not well-recognised. Here we describe the implementation of a post-COVID clinic in an Irish tertiary centre after the first wave of the pandemic. This study describes the characteristics of our patient cohort and the operations and outcomes of the clinic, exploring some of the risk factors for developing post-COVID syndrome and the appropriateness of the triage system employed.

**Methods:**

All SARS-CoV-2 positive patients from March 10^th^ to June 14^th^ 2020 were telephone-triaged as red, amber or green based on ongoing symptoms with clinic appointments scheduled accordingly. All clinic visits were face-to-face with the infectious diseases medical team and a proforma for each patient was completed. Data were collected retrospectively by reviewing the proformas and the electronic medical record (EMR).

**Results:**

311 patients attended the clinic. Median time from illness to clinic appointment was 95 days (IQR 77–105.5). 204 patients (66%) were female, 192 (62%) were hospital staff, and the median age was 43 years (IQR 31–53). 138 patients (44%) had required hospital admission. At their first clinic visit 219 patients (70%) had ongoing symptoms. A further appointment was made for 62 patients (20%). 34 patients (11%) were discussed at an MDT meeting, and 55 (18%) were referred onward to a specialist service. 85% of those triaged green, 73% of those triaged amber, and 39% of those triaged red did not receive further follow up after one clinic visit. Patients were more likely to require follow up with reported dyspnoea (OR 5.6; 95% CI 2.8–11.3; p <0.001), cough (OR 3.0; 95% CI 1.1–8.4, p = 0.04), and palpitations (OR 3.6; 95% CI 1.0–12.3; p = 0.04). Female sex was associated with increased odds of a higher triage category (OR 1.8; 95% CI 1.08 to 3.20; p = 0.02), as was requiring admission to hospital (OR 4.0; 95% CI 2.34 to 6.90; p < 0.001).

**Conclusion:**

The long-term effects of COVID-19 are significant with 70% of our cohort experiencing persistent symptoms. Persistent dyspnoea, cough and palpitations were associated with increased need for follow up. This study also suggests that a traffic light telephone-triage service followed by a face-to-face medical-led clinic could be an effective way of identifying patients who require further management.

## Introduction

COVID-19, caused by severe acute respiratory syndrome coronavirus 2 (SARS-CoV-2) infection, has had a huge global impact since its recognition in late 2019. In January 2020 the WHO declared COVID-19 a public health emergency; by March a pandemic had been declared [1]. As of November 2022, there has been over 600 million documented infections and over 6 million deaths [2].

While a vast amount of research has been conducted on COVID-19 to date, the predominant focus has been on the acute phase of the illness; particularly the epidemiology, clinical features, prognosis, immunology and potential therapeutics [3, 4]. COVID-19 is a systemic disease and although it predominantly affects the respiratory system [5], disturbances in the cardiovascular, coagulation and gastrointestinal systems have been described [6].

There is significant interest in so-called post-COVID syndrome or ‘long COVID’, a spectrum of variable symptoms and signs following initial infection. It appears to be a multisystem condition, sometimes occurring after a relatively mild initial illness [7, 8]. Post-COVID syndrome is defined by the National Institute for Health and Care Excellence (NICE) as ‘signs and symptoms that develop during or after an infection consistent with COVID-19 which continue for more than 12 weeks and are not explained by an alternative diagnosis’ [9]. The estimated incidence of post-COVID syndrome is significant with one study reporting 57% of patients having at least one feature of long-COVID 6 months post initial illness [10].

This novel condition presents many challenges given its multisystem nature and variable correlation with the severity of initial illness. The underlying pathophysiology of the condition remains unclear, and there are no proven directed therapies. There is a small, evolving evidence base but there is no standardised screening or management framework [9]. However, a multidisciplinary approach has been widely recommended for these patients [9, 11]. Further research is merited to establish clear screening and diagnostic criteria, management guidelines and follow up pathways.

The purpose of this study was to determine the clinical characteristics of patients who attended the post-COVID clinic in our hospital and the long-term effects of their SARS-CoV-2 infection, as well as exploring some of the risk factors for developing the condition and the appropriateness of the triage system employed. We also aim to describe the operations and outcomes of this new clinic which was established in a large tertiary referral hospital in response to the evolving healthcare needs associated with the pandemic.

## Study methodology

### Ethics statement

This study was approved by the MMUH institutional review board (reference 1/378/2221 TMR). Consent was waived for this study due to the full anonymisation of data.

### Clinic background

The Mater Misericordiae University Hospital (MMUH) established a Post-COVID Clinic in June 2020 to follow up all patients who tested positive for SARS-CoV-2 on polymerase chain reaction (PCR) testing through hospital services during the first wave of the pandemic (between March 10^th^ and June 14^th^ 2020). The majority of these patients were tested and diagnosed through the hospital, did not meet criteria for admission, and were monitored remotely via a COVID virtual clinic (CVC) using SpO_2_ readings, heart rate readings and dyspnoea scores [12]. The rest of the clinic cohort encompassed those that were admitted to hospital with COVID-19 and those with hospital acquired COVID-19.

The purpose of the post-COVID clinic was to review all patients after their acute illness and to screen for potential sequelae of their SARS-CoV-2 infection. It was conceived as a follow-up service that would rule out acute or life-threatening complications, to determine if persistent symptoms were caused by post-COVID syndrome or a new, unrelated diagnosis, and refer on to a specialist service for ongoing care if needed. It provided direct access to the wider multidisciplinary team, reducing service duplication and waiting times.

#### Triage process

The CVC team contacted patients by phone and assessed their current symptoms as red (chest pain, shortness of breath at rest or on minimal exertion), amber (all other symptoms including shortness of breath on exertion) or green (no symptoms). Follow up outpatient appointments were scheduled on the basis of their triage category.

Those triaged as red were contacted the same day by an infectious diseases registrar for a secondary phone assessment to determine the requirement for immediate review, next day assessment or assessment within 1 week. Those identified as amber were offered an outpatient appointment within 8 weeks. Those classified as green did not automatically receive an outpatient appointment, and were accommodated as service constraints permitted, or if a strong preference was expressed by the patient in their interaction with the service.

#### Clinic visit

**Fig 1:**
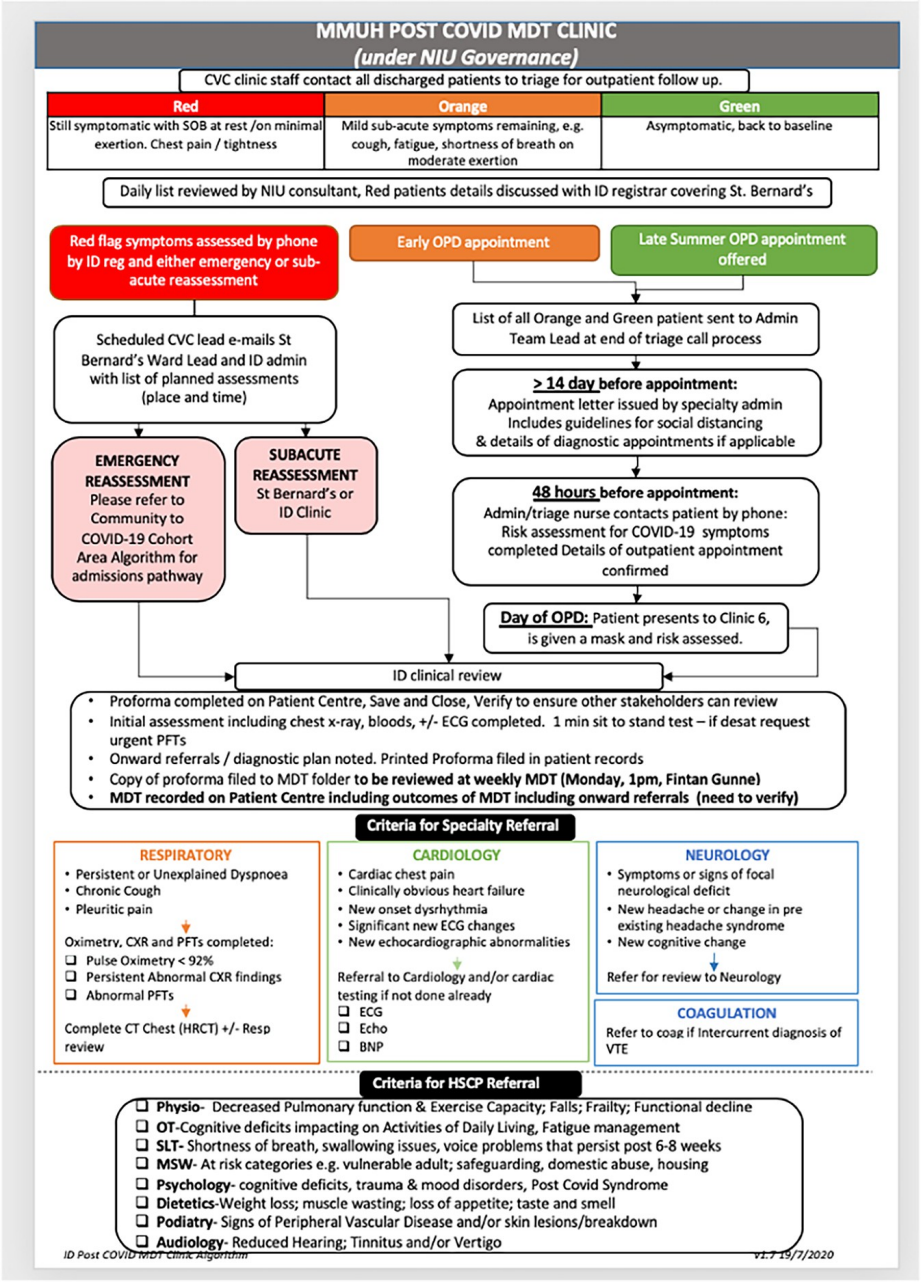
Post-COVID MDT clinic algorithm (Version 1.7, dated 19/07/2020).

All clinic visits were face to face visits with a member of the infectious diseases medical team, with oversight by an infectious diseases consultant. A proforma was developed to document symptoms, diagnostics requested and follow up plans at each patient’s first post-COVID clinic visit. A Post-COVID multidisciplinary team (MDT) algorithm was developed to inform the triage system and first clinic attendance, specifically detailing when to consider further investigations [Fig 1].

#### Multidisciplinary meeting

Given the multisystem nature of post-COVID syndrome the Mater hospital also established a post-COVID MDT meeting to run in conjunction with the post-COVID clinic. Four post-COVID MDT meetings were held over July and August 2020; attended by the infectious diseases and respiratory medical teams, clinical psychology, physiotherapy, occupational therapy, and medical social work. The main criteria for MDT discussion were patients with significant or unusual symptoms, patients whose symptoms did not fulfil the clinic algorithm pathway, and patients requiring psychology or medical social work input. Decisions were made regarding the utility of further post-COVID clinic appointments, investigations and referrals. All investigations and referrals were requested via our EMR, regardless of whether patients were discussed at these meetings or not.

### Study objectives

The objective of this study was to determine the characteristics of patients reviewed in our post-COVID clinic and to document their long-term outcomes. Additionally, we aimed to describe the operations and outcomes of a new post-COVID clinic in a large tertiary referral hospital with a high incidence of COVID-19, analysing also the some of the risk factors for developing the condition and the suitability of the triage system employed.

### Study design

This was a retrospective study of anonymised patient data. Data were collected from proformas that were filled out at every patient’s first visit to the post-COVID clinic and from our institution’s EMR.

Data collected included demographic data, clinical data from the time of their initial COVID-19 illness, clinical assessments from the time of their first clinic visit, further investigations ordered and specialist referrals made, and MDT meeting outcomes. Any investigation ordered or specialist referral made from the clinic was as part of the standard of care for enrolled patients and not for research purposes.

#### Study population

All patients who were living independently and had been treated by MMUH for COVID-19 in the first wave of the pandemic (between March 10^th^ and June 14^th^ 2020) and who had been triaged for the post-COVID clinic were included. This included patients admitted to the hospital and those monitored remotely via our COVID virtual clinic.

Patients with COVID-19 who were discharged to residential homes or nursing homes were not included in the phone triage on the basis of perceived difficulties contacting patients, logistic and infection prevention and control issues with arranging transfers to the clinic from residential homes. Similarly, it was anticipated that, unlike community-based patients who anecdotally have limited access to primary care, their general practitioner (GP) or Medicine for the Elderly consultant with clinical governance for their care would manage them locally.

#### Statistical analysis

All analysis was carried out using IBM SPSS Statistics for Windows, Version 26. Data is presented as n (%) or median (interquartile range (IQR)) unless otherwise stated. Missing data values are reported in the analysis. χ^2^ test was used for categorical variables and the Mann-Whitney U test was used for non-parametric continuous variables. An ordinal regression model was used to test whether patients’ baseline characteristics or features of their index illness predicted their subsequent triage category at follow up, with α set at 0.05, 95% confidence intervals (CIs) and odds ratios (ORs) calculated. Binary logistic regression was used to correlate likelihood of requiring follow up with individual reported post-COVID symptoms.

## Results

**Fig 2:**
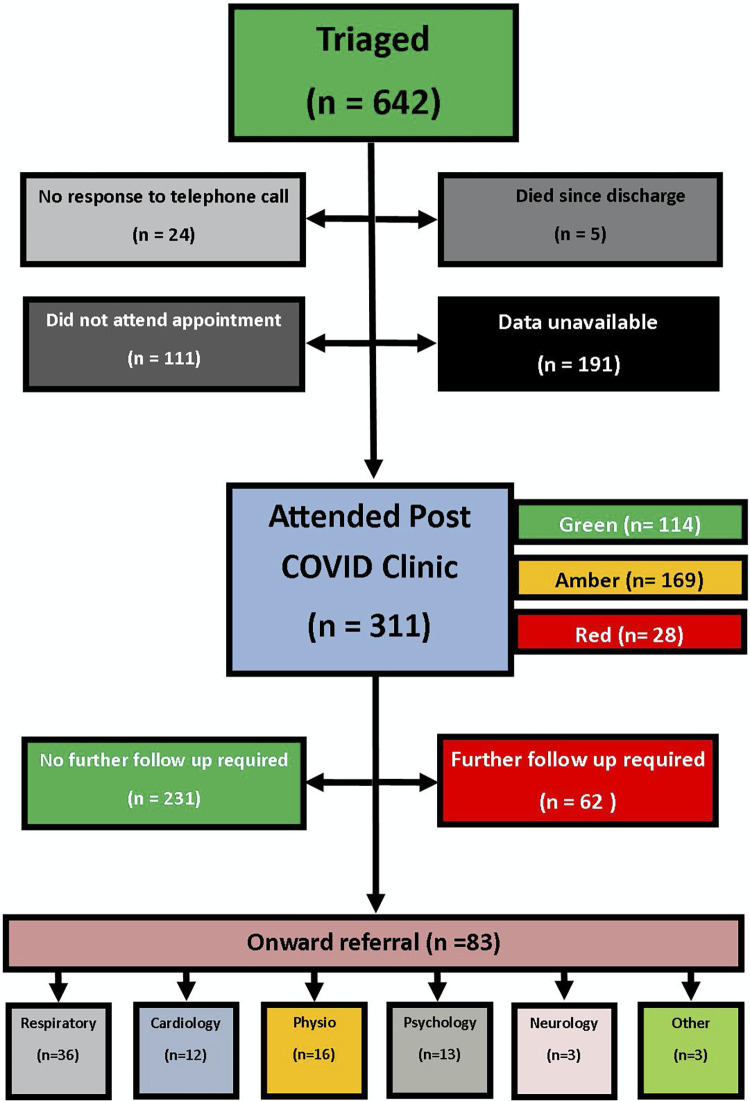
Flow chart of patients from initial triage to onward referral.

Six-hundred and forty-two patients were triaged for outpatient post-COVID clinic appointments. There was no response from 24 patients, and 5 patients had died since discharge. The cause of death of these patients was not recorded. Of the patients triaged, 311 patients attended their post-COVID clinic appointment and 111 failed to attend. A further 191 patients had no data available as no appointment was offered or there was no proforma on the EMR available [Fig 2].

Of the 311 patients that attended our post-COVID clinic, 114 (37%) had been triaged as green, 169 (54%) as amber and 28 (9%) as red. The median time from positive PCR test to first clinic appointment was 95 days (IQR 77–105.5). The median age was 43 years (IQR 31–53), 204 (66%) were female and 192 (62%) were healthcare workers.

Of those who attended, 138 patients (44%) had required hospital admission during their index COVID illness. The remaining 173 patients had been managed remotely by our CVC. Of the 138 patients admitted, 17 (12%) had required admission to critical care (intensive care unit (ICU) or high dependency unit (HDU)) and 69 (50%) had required supplemental oxygen therapy. Fifty-one patients (37%) had required supplemental oxygen via nasal prongs or venturi mask, 9 (6.5%) had required high flow nasal oxygen or non-invasive ventilation, and 9 (6.5%) had required invasive ventilation. Overall, the median length of admission was 6 days (IQR 2–10).

At their initial post-COVID clinic visit 221 of 311 patients (71%) had ongoing symptoms. The most common symptoms were dyspnoea (30%) and fatigue (33%), followed by chest pain (13%), cough (9%) and loss of taste or smell (9%). Symptoms were not documented for 6 patients (2%). Twenty-four patients (8%) had not returned to work and 22 (7%) reported mood disturbance [Table 1].

**Table 1:** Symptoms at 1^st^ clinic visit.

Symptoms	n (%)
Cough	28 (9%)
SOB	92 (30%)
Fatigue	102 (33%)
Chest pain	39 (13%)
Anosmia/loss of taste	27 (9%)
Palpitations	13 (4%)
Headache	18 (6%)
Mood disturbance	22 (7%)
Arthralgia	17 (5%)

There were a number of clinical investigations performed or requested at the first clinic visit. The most frequent investigations performed included laboratory blood tests (231 patients, 74%), chest plain film radiograph (256 patients, 82%) and ECG (28 patients, 9%). Computed tomography (CT) of the thorax was requested for 11 patients (4%), pulmonary function tests (PFTs) were requested for 60 patients (19%), 23 (7%) had an echocardiogram requested, and 11 (4%) were booked for a 24-hour holter monitor. The referral rate of patients to other specialist services was low at the first clinic visit with 10 (3%) patients referred to the respiratory service, 8 (3%) patients referred to cardiology, 7 (2%) patients referred to our psychology service and 7 (2%) to physiotherapy. Further post-COVID clinic follow up was arranged for 62 (20%) patients and 34 (11%) patients were deemed to require discussion at the COVID MDT meeting. The majority of patients attending the post-COVID clinic (231 patients, 74%) did not require further follow up or MDT discussion, and were discharged from the clinic after one visit.

We analysed the likelihood of requiring follow up with individual reported post-COVID symptoms. Patients were more likely to require follow up with reported dyspnoea (OR 5.6; 95% CI 2.8–11.3; p <0.001), cough (OR 3.0; 95% CI 1.1–8.4; p = 0.04), and palpitations (OR 3.6; 95% CI 1.0–12.3; p = 0.04). Patients were more likely to require MDT discussion in the event of on-going dyspnoea (OR 4.8; 95% CI 2.1–10.7; p < 0.001) or cough (OR 4.8; 95% CI 1.7–13.5; p = 0.003).

At subsequent post-COVID clinic follow up, there were 25 additional referrals to the respiratory service, 8 referrals to physiotherapy, 6 to psychology, 3 to cardiology, 2 to neurology, 1 to haematology and 1 to dermatology. Total referrals to each specialty are outlined in Fig 2.

## Further analysis by triage category

There were 114 (37%) patients triaged as green, 30 (26%) of which had required hospital admission. Of those admitted, 4 (4%) had required admission to critical care. Of those triaged green, 85% did not receive any form of further follow up after one clinic visit. Of the 169 (54%) patients triaged as amber, 88 (52%) had required hospital admission and thirteen (8%) had required a critical care admission. Of this group, 73% did not receive any form of further follow up after one clinic visit. Lastly, of the 28 (9%) patients who were triaged as red, 22 (79%) had required hospital admission. There were no admissions to critical care but oxygen therapy had been administered to 8 (29%) patients. Eleven (39%) of those triaged red did not receive any form of follow up after one clinic visit. Overall a slightly greater proportion of those in the amber or red category required follow up appointments rather than those assigned to the green triage category (19.3% versus 11.4%; one-sided p = 0.047) [Tables 2 and 3].

**Table 2:** Baseline characteristics and COVID-19 severity by triage category.

Patient Baseline Characteristics	Total (n = 311)	Green (n = 114)	Amber (n = 169)	Red (n = 28)	p
**Age (Years): median [IQR]**	43 [31,53]	37 [30,50]	44 [32,54]	48 [42, 53]	0.02
**Sex (Female): n (%)**	204 (66)	72 (63)	113 (67)	19 (68)	0.79
**Staff: n (%)**	192 (62)	85 (75)	97 (57)	10 (36)	<0.001
**Interval between initial positive test result and clinic (days)**	94 (77–106)	102 [96–118)	82 [70–99]	72 [63–85]	<0.001
**Co-morbid conditions**					
Chronic airways disease n (%)	51 (16.4)	14 (10)	29 (17.2)	8 (29)	0.105
Diabetes: n (%)	24 (8)	10 (9)	9 (5)	1 (4)	0.87
HTN: n (%)	52 (17)	18 (16)	27 (16)	6 (21)	0.5
IHD: n (%)	11 (4)	3 (3)	5 (3)	2 (7)	0.5
Obesity: n (%)	9 (3)	4 (3.5)	2 (1)	3 (11)	0.28
Current or ex-smoker: n (%)	9 (3)	1 (1)	5 (3)	2 (7)	0.13
**Severity of COVID-19 illness**					
Asymptomatic: n (%)	8 (3)	5 (4)	3 (2)	0 (0)	0.29
Hospital Admission: n (%)	138 (44)	30 (26)	88 (52)	22 (79.0)	
Length of admission (Days): median [IQR]	6 [2,10]	5 [2, 9]	7 [2, 13]	5 [3, 78]	0.04
HDU admission: n (%)	5 (2)	3 (3)	2 (1)	0 (0)	0.5
ICU admission: n (%)	12 (4)	1 (1)	11 (7)	0 (0)	0.03
Required supplemental O2 during admission n (%)	70 (23)	15 (13)	47 (28)	8 (29)	0.01
Required NIV/HFNO during admission n (%)	10 (3)	4 (4)	6 (4)	0 (0)	0.6

**Table 3:** Clinical outcomes by triage category.

Clinic Outcomes	Total (N = 311)	Green (N = 114)	Amber (N = 169)	Red (N = 28)
MDT discussion: n (%)	34 (11%)	5 (4%)	15 (9%)	14 (50%)
Further Post-COVID clinic follow-up: n (%)	62 (20%)	14 (12%)	41 (24%)	7 (25%)
Patients requiring onward referral: n (%)	56 (18%)	10 (9%)	30 (18%)	16 (57%)
Discharged after 1 visit: n (%)	231 (74%)	97 (85%)	123 (73%)	11 (39%)

An ordinal regression model was used to test if patients’ baseline characteristics or features of their index illness predicted their subsequent triage category at follow up. The overall regression was statistically significant (pseudo R^2^ = 0.076, p < 0.001). Female sex was associated with increased odds of a higher triage category (OR 1.8; 95% CI 1.08 to 3.20; p = 0.02), as was requiring admission to hospital (OR 4.0; 95% CI 2.34 to 6.90; p < 0.001). No significant relationship between age or presence of any baseline chronic condition (chronic airways disease, hypertension, diabetes mellitus or ischaemic heart disease) was noted. A separate model analysing admitted patients found no relationship between surrogate markers of disease severity and subsequent triage category, i.e. requirement of supplemental oxygen, non-invasive ventilation, length of stay, or HDU/ICU admission. As per Table 2 there does appear to be significantly more patients in the amber group who required supplemental O2 and ICU admission but no statistically significant association was borne out when multivariate analysis was performed.

## Discussion

Our study demonstrates several key findings. Firstly it is clear that the long-term effects of COVID-19 are significant with 70% of our cohort experiencing ongoing symptoms at time of first clinic visit. Secondly that there is a need for specialist post-COVID clinics with multidisciplinary involvement given the broad spectrum of specialist referrals generated from our clinic. Thirdly this study suggests that using a traffic light telephone-triage system is an effective way of identifying those patients who are most in need of timely follow up.

### High burden of persistent symptoms

This study demonstrates the significant proportion of patients who have persistent symptoms several months after their initial SARS-CoV-2 infection and who required repeated follow up appointments. The most common symptoms described in our patient population were fatigue, dyspnoea, chest pain, cough, and altered smell and taste. This is consistent with data from other studies. Huang et al. described a cohort of 1733 patients who had previously been infected with SARS-CoV-2. Six months post infection, 76% of patients reported at least one persistent symptom, with the most common symptoms being fatigue, muscle weakness and difficulties with sleep [13]. Bellan et al. also described common persistent symptoms seen in their study cohort 4 months after initial illness with one third of patients describing persistent arthralgia and myalgia [5]. Interestingly, our cohort reported a much lower rate of these symptoms with approximately 5% describing arthralgia, demonstrating the wide variation in post-COVID symptoms.

A significant proportion of our cohort remained out of work (8%) or disclosed ongoing alterations of mood (7%). It is speculated that overall quality of life may be impacted by post-COVID syndrome, although this was not directly measured. The link between post-COVID syndrome and adverse psychological outcomes has been well described in the literature [14]. Troyer et al.’s article published in April 2020 predicted a high burden of neuropsychiatric consequences of SARS-CoV-2 infection and emphasised the vital need for close monitoring of such symptoms in those exposed to the virus [15]. One retrospective cohort study of over 200,000 patients demonstrated significant rates of neurological and psychiatric diagnoses in the 6 months post COVID-19 diagnosis with an estimated incidence of 33% [16]. A recent meta-analysis reported anxiety and depression rates of 23% and 12% respectively amongst patients with post-COVID syndrome [17].

Of note there was a large volume of non-attendance to the clinic which may introduce bias into these results. Of the 110 patients who failed to attend, 57 had been triaged as green, 52 triaged as amber and 1 triaged as red. All those triaged as green had returned to baseline and were asymptomatic at time of triage phone call. Data is unavailable regarding the clinical status of the amber or red patients. It is possible that the patients who didn’t attend the clinic were those with milder symptoms leading to the cohort being overrepresented with symptomatic patients. The exact reasons for clinic non-attendance however are not known.

With regards to the 191 patients who had no data available, 9 of these patients had been triaged as amber but unfortunately the clinic proforma was not available on EMR. The other 182 patients were triaged as green and were not offered an appointment due to clinic constraints. Assuming the majority of these patients were asymptomatic or had mild symptoms, this is also contributing to the overrepresentation of symptomatic patients within the analysed cohort.

### Multidisciplinary approach

It is clear that patients with post-COVID syndrome have heterogenous symptoms but the underlying pathophysiology of the syndrome remains poorly understood. It has been recommended by several organisations [7, 18, 19] that patients with COVID-19 should have some form of follow up care, particularly if they had required hospitalisation or if they have new or persistent symptoms. Individuals discharged from hospital after acute COVID-19 have been shown to have an increased risk of mortality, readmission, and multiorgan dysfunction compared with the general population [20].

The main approach of post-COVID care at present is to rule out acute or life-threatening complications and to determine if persistent symptoms are caused by post-COVID syndrome or a new, unrelated diagnosis. Once these initial investigations are carried out the mainstay of treatment is multidisciplinary supportive care [18].

The multidisciplinary approach has been shown to be crucial in post-COVID care given the heterogenous nature of post-COVID syndrome [11]. The patients in our cohort reported a broad spectrum of symptoms generating a large volume and spread of investigations and specialist referrals, with referrals made to respiratory, cardiology, psychology, physiotherapy, neurology, haematology, and dermatology. The clinic provided streamlined access to the multidisciplinary team through our MDT meeting.

### Our clinic model

Despite the recommendation to establish post-COVID care pathways, there is no standardised framework to create or implement these pathways. NHS England recommend hospitalised patients should have a 12 week post hospital discharge assessment and those admitted to critical care should have a multidisciplinary clinic assessment at 4–6 weeks post discharge. Those managed in primary care should seek referral to a post-COVID assessment clinic through their GP if they have persistent or new symptoms [18].

Our clinic model consisted of a nurse-led telephone triage service followed by a medical-led face to face clinical assessment. The triaging service we developed appears to have been effective with those triaged as red generating the most investigations and onward referrals and those triaged as green most likely to be discharged from the clinic after one visit. This may help other healthcare settings in determining which patients merit referral to a specialist post-COVID clinic. Moreover, it may allow determination and planning of resources required to maintain operation of a multidisciplinary post-COVID clinic, dependent on the average numbers of patients referred with red triage category symptoms. A study by Menges et al found an association between need for follow up and a number of factors such as initial hospitalisation, age > 40 years, female sex, and symptoms of dyspnoea, fatigue, and depression [21]. This was reflected in our cohort with female sex and hospitalisation both associated with higher odds of a higher triage category, however we did not find any other statistically significant associations.

Interestingly, the severity of our patient’s post-COVID syndrome did not necessarily correlate with the severity of their initial illness. For example, none of the patients who were triaged as red had required admission to critical care and we found no relationship between surrogate markers of disease severity and subsequent triage category. This highlights the importance of devising a system that can identify patients with severe or persistent symptoms instead of basing the need for follow up care solely on the severity of initial COVID-19 illness.

### Limitations

The large volume of non-attendance to the clinic is a potential source of bias to the study. For instance, those that felt well may have not felt the need to attend our service, therefore over representing the cohort with symptomatic patients. Secondly, the relatively high proportion of healthcare workers undermines the generalisability of our findings to the greater population, with staff members, including those in the green triage category, likely having greater access to the clinic and therefore occupying the majority of that group. While genomic sequencing data for our cohort was not available, on the basis of the timing of our recruitment we assume that our cohort was infected exclusively with the original Wuhan variant of SARS-CoV-2, which has been subsequently replaced with the Alpha (B.1.1.7), Delta (B.1.617.2) and Omicron strains of the virus, amongst others. It remains unclear how the presentation of post-COVID syndrome will differ in individuals infected with these newer variants [22]. Lastly, we did not capture the post-COVID course of our elderly patient population living in residential care. A study with a longer follow up period, encompassing the healthcare sought by participants in other settings, and which assessed the alternative diagnoses for patients’ symptoms would also provide insight into the reliability and necessity of the investigations performed by our service, however this was beyond the scope of this paper.

## Conclusion

The burden of post-COVID symptoms are significant with 70% of our cohort experiencing persistent symptoms. Persistent dyspnoea, cough and palpitations were associated with increased need for post-COVID follow up care. We found no association between the severity of initial illness and the severity of post-COVID symptoms, highlighting the difficulty of detecting patients most in need of follow up care. This study describes the implementation of a specialist multidisciplinary post-COVID clinic, and we suggest that a traffic light telephone-triage service followed by a face-to-face medical-led review could be an effective way of identifying these patients. Longer follow-up studies in a larger population are necessary to understand the full spectrum and duration of health consequences from COVID-19.

## Supporting information

S1 Dataset (XLSX)
